# Assessment of the changes in growth, photosynthetic traits and gene expression in *Cynodon dactylon* against drought stress

**DOI:** 10.1186/s12870-024-04896-x

**Published:** 2024-04-02

**Authors:** Maryam Noor, Jibiao Fan, Muhammad Kaleem, Muhammad Tanveer Akhtar, Shixuan Jin, Usman Nazir, Chuan-Jie Zhang, Xuebing Yan

**Affiliations:** 1 College of Animal Science and Technology, Yangzhou University, Yangzhou, 225009 China; 2 Department of Botany, University of Agriculture, Faisalabad, 38040 Pakistan; 3 College of Horticulture and landscape architecture, Yangzhou University, Yangzhou, 225009 China

**Keywords:** Bermudagrass, Drought stress, Tolerance, Gene expression

## Abstract

Drought stress considered a key restrictive factor for a warm-season bermudagrass growth during summers in China. Genotypic variation against drought stress exists among bermudagrass (*Cynodon sp*.), but the selection of highly drought-tolerant germplasm is important for its growth in limited water regions and for future breeding. Our study aimed to investigate the most tolerant bermudagrass germplasm among thirteen, along latitude and longitudinal gradient under a well-watered and drought stress condition. Current study included high drought-resistant germplasm, “Tianshui” and “Linxiang”, and drought-sensitive cultivars; “Zhengzhou” and “Cixian” under drought treatments along longitude and latitudinal gradients, respectively. Under water deficit conditions, the tolerant genotypes showed over-expression of a dehydrin gene *cdDHN4*, antioxidant genes *Cu/ZnSOD* and *APX* which leads to higher antioxidant activities to scavenge the excessive reactive oxygen species and minimizing the membrane damage. It helps in maintenance of cell membrane permeability and osmotic adjustment by producing organic osmolytes. Proline an osmolyte has the ability to keep osmotic water potential and water use efficiency high via stomatal conductance and maintain transpiration rate. It leads to optimum CO_2_ assimilation rate, high chlorophyll contents for photosynthesis and elongation of leaf mesophyll, palisade and thick spongy cells. Consequently, it results in elongation of leaf length, stolon and internode length; plant height and deep rooting system. The *CdDHN4* gene highly expressed in “Tianshui” and “Youxian”, *Cu/ZnSOD* gene in “Tianshui” and “Linxiang” and *APX* gene in “Shanxian” and “Linxiang”. The genotypes “Zhongshan” and “Xiaochang” showed no gene expression under water deficit conditions. Our results indicate that turfgrass show morphological modifications firstly when subjected to drought stress; however the gene expression is directly associated and crucial for drought tolerance in bermudagrass. Hence, current research has provided excellent germplasm of drought tolerant bermudagrass for physiological and molecular study and future breeding.

## Introduction

Agriculture water demand will be double in 2050, so the accessible fresh water is expected to drop by 50%, because of climatic changes [1]. A growing challenge for the turf grasses growing in arid or semi-arid zones is inadequate water availability for basic irrigation [2]. Improper irrigation called drought stress causes, intercellular and intracellular water loss which limits turfgrass growth and development [3]. Drought stress is a world’s growing problem.

Drought induces morphological changes in grass such as reduced and vegetative growth and leaf length, and the physiological indicators such as the reduced photosynthetic rate, water use efficiency and stomatal conductance, and finally modify the anatomical structure of stem and root [4]. The water deficit conditions results in reactive oxygen species (ROS) overproduction, which results in irreversible cell damage and limitation in photosynthesis and ultimately reduction in chlorophyll *a*, *b* and total chlorophyll contents [5].

However, drought tolerance is actually a complex attribute depends upon stress period, severity and plant developmental phase [6]. Different grasses develop varied mechanism to avoid, escape and tolerate against drought [7]. A valuable germplasm for understanding drought tolerance mechanisms, are a number of perennial grass species because of their wide genetic variation [8].

Thus, the study on drought resistance procedures is becoming essential for turfgrass managers and breeders. Knowledge of drought resistance among turfgrass genotypes has vital role for selecting grasses that persist during drought conditions.

Currently, to manage with inadequate water supply special attentions repaid to mechanisms of grass, such as physiological and biochemical metabolisms, cross-talks between several hormones, gene expression regulation and proteomic profiling. Further, it aids in development of varied genetic approaches for improvement of grass drought tolerance [9]. Comparatively, *C. dactylon* (bermudagrass) is an important drought tolerant turfgrass [10].

Bermudagrass (*Cynodon dactylon* [L.] Pers.) is a member of genus *Cynodon* and family *Poaceae* [11]. A widely distributed grass worldwide between 45°N and 45°S [12]. *Cynodon* spp. is normally found in transition zones where it provides an excellent turf surface and ground cover like appearance used for turf, forage, soil stabilization, golf course, fairways and athletic fields as well [13]. Analysis of natural variations against drought stress tolerance revealed that a great variation was found within different bermudagrass species against stress [10].

Its mechanisms against drought include, drought avoidance and tolerance [14]. Some bermudagrass have morphological attributes like deep and shallow rooting system, thick cuticula and lesser stomata, which may enhance its drought avoidance [15]. The others, can develop physio-biochemical mechanisms during internal water deficit conditions [3]. Bermudagrass are classified as semi-tolerant to highly-tolerant towards drought [16]. In addition, plant responses against environmental stresses are under transcriptional control of several stress-related genes upregulation or downregulation which results in a reaction to stimuli [17].

This study aimed to observe the differences in gene expression of thirteen bermudagrass germplasm against drought stress and to study possible processes taking part in drought stress tolerance. Bermudagrass germplasm were collected from different latitude and longitudes of china. The natural modifications against drought were evaluated. The interactions between drought tolerances and a number of indicators were further discussed and compared. These findings provided certain insights to better understand the genetic basis of bermudagrass against water deficit.

## Materials and methods

### Collection sites

Total 13 genotypes of bermudagrass collected from latitude gradients 34°32′43” N and longitude gradients 105°57′34” E of China. Total annual mean temperature and annual average precipitation is also provided (Table 1).

**Table 1:** *C. dactylon* populations collected from different latitudes and longitude in China

Gradient	Population code	Localities	Latitude(N)	Longitude(E)	Altitude/m	Annual average temperature/°C	Annual average precipitation/mm
Longitude gradients	1	Tianshui	34°32’43”	105°57’34”	1050	11.4	500.7
	2	Baoji	34°21’54”	107°41’03”	630	13.5	645.9
	3	Sanmenxia	34°42’29”	111°03’49”	340	14	558.1
	4	Zhengzhou	34°54’04”	113°38’20”	90	14.7	640.8
	5	Shanxian	34°46’31”	116°09’11”	30	14.2	621.4
	6	Zaozhuang	34°38’48”	117°49’20”	89	14.4	820.3
	7	Lianyungang	34°46’09”	119°27’06”	50	14.5	883.9
Latitude gradients	8	Zhongshan	22°35’40”	113°23’17”	0	22.0	1846.8
	9	Youxian	27°00’59”	113°23’07”	342	18.1	1518.4
	10	Linxiang	29°28’32”	113°26’48”	45	16.8	1582.5
	11	Xiaochang	31°18’59”	114°02’15”	30	16.8	1138.0
	12	Zhumadian	33°09’47”	114°03’45”	85	15.2	990.4
	13	Cixian	36°18’40”	114°11’51”	107	13.4	509.2

### Plant materials establishment and experimental conditions

In the following experiment 3–4 cm stolons of 13 bermudagrass genotypes were planted on 9th of May 2023 in plastic pots (diameter 16 cm, height 17.5 cm) filled with clay loam soil and yellow sand with a ratio of 1:1 in the greenhouse of Yangzhou University Jiangsu China at 25 °C with a 12/12 h photoperiod (light intensity 10,000–16,000 lx) for 40 days to establish the bermudagrass plant. The experiment was designed as complete randomized block design with three replications of each treatment of each genotype. The established plants were transferred outside of the greenhouse under direct sunlight for drought treatments.

### Drought stress imposition

To estimate the field capacity (FC) of the pots, two perforated pots, filled with oven-dried potting mix, were saturated with tap water and then allowed to drain for six hours. Following this, 250 g of potting mix was sampled from the center of each pot to record the wet mass. Samples were then oven-dried at 70 °C for 72 h to determine the dry mass of sampled potting mix. The gravimetric water contents (%) of the potting mix was measured using the equation-1 [18];

1 $$\begin{array}{*{20}{c}}{{\text{Gravimetric}}\,{\text{water}}\,{\text{contents}}\,(\% ) = \frac{{{\text{Wetmass}} - {\text{Drymass}}}}{{{\text{Drymass}}}} \times 100} \end{array}$$

All pots were irrigated twice a week with 85% FC to prevent excessive water drainage. At this stage, plants were treated with two watering regimes, Control (normal irrigation) and drought stress treatment (DS, drought stress only), where soil water content (SWC) was maintained at 50% FC and control (absolute control where SWC was maintained at 85% FC. Before DS application, irrigation was gradually reduced for five days for acclimation to attain 50% FC. During the experiment, pots were reshuffled manually after every 2nd week to minimize pseudo growth and border effects.

After application of 28 days of drought stress the morphological parameters were observed and plant material was saved for further analysis.

### Statistical analysis and design

The current experimentation was designed as a completely randomized design with three replications and two treatments to screen out the drought tolerance in *Cynodon dactylon*. The statistical interpretations were validated by R statistical software (R Core Team, 2021) under an R R-integrated environment (R Studio Team, 2021). The replicate values are subjected to a two-way analysis of variance at *p* ≤ 0.05 to evaluate the difference between varieties under control and drought applications. To compare the means and SE of the means Tukey’s HSD was used through the “agricolae” package of R software (Mendiburu, 2020). The ellipsed PCA was done by using the package “FactoMineR and ggplot2” by R software. The correlation matrix was computed by the ggbiplot2 package. The clustered heatmap was made by using the R customized code “pheatmap” and the hierarchical dendrogram was constructed by the &quot;factoextra&quot; package.

### Measurement of growth parameters

The morphological parameters for each bermudagrass genotype was examined include plant height, stolon length, leaf length, width of leaf and number of tillers. Plant height (cm) was measured with measuring tape. Stolon length (mm) was measured with vernier caliper by selecting the random stolon from the whole pot. For leaf length and width (mm), the fully expanded third leaf from the apical meristem was selected, measured with vernier caliper and was averaged by four replicates. Additionally, number of tillers was counted quantitatively according to the method of [19]. At the last day of experiment the fresh weight was obtained and then oven dried at 80 °C for 8 h until constant value, for the dry weight measurement.

### Assessment of relative water content (RWC)

For assessment of relative water content of leaf the method of [20] was followed, the fully expended forth leaf was detached and measured the weight on the weighing balance in the same greenhouse. FW (fresh weight) of the samples were quantified every 1 h intervals till 8 h, then immersed in water for 3–4 h until the weight of leaves was constant. The turgid weight as (TW) was measured. The dry weight (DW) was determined after 16 h incubation at 80 °C. RWC was measured by using the following formula:

$${\text{RWC}}\,\left( \% \right)\, = \left( {{\text{FW}} - {\text{DW}}} \right)/\left( {{\text{TW}} - {\text{DW}}} \right) \times 100$$

### Quantification of electrolyte leakage (EL)

For the EL assay [21] method was applied, in short 0.1 g leaves were rinsed with distilled water and immersed in 10 ml of double deionized water. The mixture was then shaken at room temperature for 6 h to ensure that the sample and water mixed thoroughly. The initial conductivity (Ci) of the sample was measured by using a conductivity meter (Leici-DDS-307 A, Shanghai, China). The sample was then boiled for 20 min and left to cool to room temperature. The conductivity of the sample was then measured again to determine the maximum conductivity (Cmax).

$${\text{Relative}}\,{\text{EL}}\,{\text{(\% )}} = \left( {{\text{Ci}}/{\text{Cmax}}} \right) \times 100.$$

### Chlorophyll content measurement

Plant leaf chlorophyll was determined by the method of [22] with slight modifications. Briefly, 0.1 g of fresh leaf samples was immersed into 1mL of 80% acetone that was contained grinding beads in 1.5mL centrifuge tubes. Then tubes were placed in grinding machine for 3 min and centrifuged at 15,000 rmp for 5 min at RT (room temperature). Absorbance of the extract was observed at 647 and 664 nm using spectrophotometer. Chlorophyll and carotenoids was calculated by using following formula.

$${\text{Chl - content}}\,\left( {*{\text{mg}}{\text{. }}{{\text{L}}^{ - 1}}} \right) = 20.2 \times {\text{OD}}647 + 8.02 \times {\text{OD}}664$$

### Photosynthetic traits

Net CO_2_ assimilation rate (*A*) and transpiration rate (*E*) of fully expanded leaves were measured using a Li Core 6400 portable photosynthesis system (Li-Core Inc, USA) according to [23] method. Photosynthetic indicators were measured in early morning (9:00–11:00 AM) under modest light conditions, PAR set at 750 µ mol^− 1^ s^− 1^ and CO_2_ at 350 µ mol^− 1^ s^− 1^.

WUE = A/E, *A* represents the net assimilation rate and *E* transpiration rate.

### Extraction of crude enzyme

0.1 g of fresh leaf sample was grounded with liquid nitrogen into fine powder. 4 ml of sodium phosphate buffer with 150 mM and pH 7.0 which was pre cooled at 4 °C was mixed in the powder. Then the mixture was shifted into 10 ml tube and centrifuged at 12000rmp and 4 °C for 20 min. The supernatant obtained at the end was the crude enzyme actually that to be determine.

### Assessment of malondialdehyde (MDA) content

The lipid peroxidation was assessed by calculating the amount of MDA as described by the protocol [24], with the thiobarbituric acid (TBA). Totally 1 mL of crude enzyme was added into 2 mL MDA reaction buffer that includes 0.6% (v/v) thiobarbituric acid (TBA) and 10% (v/v) trichloroacetic acid (TCA). The mixture was heated at temperature 95 °C for 30 min in an electric water bath and then cooled till 25 °C (room temperature) and centrifuged at 12,000 rmp at 25 °C for 10 min. The supernatant was obtained for absorbance at 450 nm, 532 and 600 nm. MDA content was calculated by using formula: (645 × (OD_532_-OD_600_) − 0.56OD_450_) × 0.015/ W.

### Measurement of H_2_O_2_ level

0.1 g fresh leaves were mixed with liquid nitrogen and grounded and then uniformly mixed with extraction buffer (50 mM sodium phosphate buffer, with pH 7.8). Then centrifuged at 12,000 rpm for 30 min at 4 °C, then 1 ml of supernatant was homogenized completely with 1 ml of 0.1% titanium sulphate in 20% H_2_SO_4_ (v/v) for 10 min. After centrifugation at 12,000 rpm for 10 min at room temperature, the absorbance of mixture was measured at 410 nm with known H_2_O_2_ concentration as standard by following the [10] procedure.

### Assessment of organic osmolytes (proline)

For the determination of proline content [25] method was followed as 0.2 g leaves were cut into small pieces and added 5 ml of sulfosalicylic acid and then kept the tubes in a water bath at 98 °C for 10 min. After cooling down at room temperature 2 ml of following plant sample mixed with 3 ml of mixture solution of 2.5% ninhydrin mixed in glacial acetic acid and phosphoric acid in 3:2 with 2 ml acetic acid then kept in a water bath at 98 °C temperature for 40 min, after normalizing at room temperature 5 ml of methylbenzene and shanked for 30 min to obtain supernatant. Finally the absorbance was checked at 520 nm with spectrophotometer.

### Quantification of cellular antioxidants

To determine the SOD activity the [10] method was followed, 0.005 mL crude enzyme extracted, was mixed with 3 mL reaction mixture which includes 2.2 ml sodium phosphate buffer (50mM, pH 7.8), 0.4849 g methionine, 0.0186 g ethylene diaminetetraacetic acid (EDTA), 0.0038 g riboflavin, 0.0153 g nitro blue tetrazolium (NBT) and 0.003 ml reaction solution without crude enzyme was set as standard. For chromogenic reaction solution, the mixture was irradiated under 4000 lx fluorescent lamp for one hour. The absorbance was calculated at 560 nm using spectrophotometer. One unit SOD activity was mentioned as amount of SOD required inhibiting NBT reduction by 50%.

The POD activity was determined by following [24], 40 µL of crude enzyme was mixed into 3 mL reaction mixture which includes sodium acetate acetic acid buffer (pH 6.0), 0.037 mL guaiacol (guaiacol was dissolved in 50% ethanol solution) and 0.056 mL 30% H_2_O_2_. Absorbance of mixture at 460 nm was increased per minute, was recorded for 3 min.

### Gene expression traits

#### RNA extraction and gene expression analysis

Total RNA of bermudagrass was executed employing Freezol Reagent kit (Nanjing Novizan Co., Ltd. China). RNA quality was determined using a NanoDrop ND-1000 spectrophotometer (NanoDrop Technologies, Rockland, DE, USA) and Bioanalyzer 2100 system (Agilent Technologies, CA, USA) were used to test the quantity and integrity of RNA samples. The first-strand complementary DNA (cDNA) was obtained with the kit (Nanjing Novizan Co., Ltd. China) by following manufacturer’s instructions. The qRT-PCR was performed via an ABI System of Fast Start Universal SYBR®Green Master Mix (Roche) using kit (Nanjing Novizan Co., Ltd. China). The primers used are listed in table (Table 2). *CdACTIN* was used as reference gene [26]. Each sample had three biological replicates.

**Table 2:** Primers used in gene expression

GENE		Primers (5 − 3)
CdHN4	F	GCGAACAGTCCGTGATAACT
	R	GACACTAATGCGCCCGGTAT
APX	F	TCCGTGAAGTAAGAGTTGTC
	R	CAGATGGGCTTGAGTGAT
Cu/Zn SOD	F	TCTTCCACCAGCATTTCC
	R	AGGCGTGGCTGAGACAAC
CdActin (Control)	F	AGGCATCCAACCAGCAGAGA
	R	ACTCAGCACATTCCAGCAGAT

Primers used for gene expression in bermudagrass under drought stress are mentioned. The forward and reverse sequence is present

## Results

### Growth traits

Under control treatment the varieties (V10, V11) showed higher PH. The SL, LL, LW and NT were higher in V12 under control and the EL was higher in V02. While, least PH and SL were assessed in V03, LL in V05, LW and NT in V08 and EL in V01 (see Table 3).

**Table 3:** Growth traits of *Cynodon dactylon* under control and drought stress

	Control						Drought					
Variety	PH	SL	LL	LW	NT	EL	PH	SL	LL	LW	NT	EL
**V01**	28.67 ± 1.00c	21.22 ± 1.78a	55.97 ± 0.97c	1.82 ± 0.09d	13.67 ± 0.57c	8.96 ± 0.26f	32.00 ± 0.33d	16.11 ± 1.47c	51.72 ± 1.23f	2.24 ± 0.12f	19.00 ± 0.88c	6.58 ± 0.34f
**V02**	22.33 ± 1.00d	13.89 ± 0.69c	39.36 ± 1.07e	2.10 ± 0.12c	7.33 ± 0.67e	23.82 ± 0.38a	39.00 ± 0.88b	15.67 ± 0.87c	47.46 ± 0.97f	2.82 ± 0.50b	18.33 ± 0.88c	14.82 ± 0.24d
**V03**	17.67 ± 1.33e	10.56 ± 0.68d	44.43 ± 1.10e	1.76 ± 0.23d	11.33 ± 1.00d	14.28 ± 0.07d	31.33 ± 0.88d	10.11 ± 0.59e	55.66 ± 0.14f	2.56 ± 0.25d	20.00 ± 0.67c	10.55 ± 0.32e
**V04**	27.00 ± 0.58c	24.56 ± 1.09a	69.43 ± 1.66b	2.38 ± 0.15b	16.67 ± 1.15b	21.00 ± 1.07a	28.00 ± 0.00d	14.56 ± 0.62d	74.02 ± 0.61c	2.58 ± 0.15d	20.00 ± 0.67c	19.48 ± 0.58b
**V05**	33.00 ± 0.88b	14.67 ± 0.38c	33.02 ± 1.47f	2.39 ± 0.15b	13.33 ± 4.63c	20.40 ± 0.04a	36.67 ± 0.58c	15.00 ± 0.51c	43.00 ± 1.20 g	2.31 ± 0.19e	28.33 ± 0.88a	15.91 ± 0.22e
**V06**	28.00 ± 0.88c	19.93 ± 0.69a	62.07 ± 0.61b	2.25 ± 0.11b	11.00 ± 0.67d	19.96 ± 0.06b	29.67 ± 0.00d	16.67 ± 0.14c	71.14 ± 0.71c	2.39 ± 0.04e	19.67 ± 0.58c	16.12 ± 0.10c
**V07**	34.67 ± 0.67b	9.93 ± 0.11e	62.14 ± 1.62b	2.40 ± 0.12b	13.67 ± 0.88c	19.87 ± 0.08b	40.33 ± 0.88b	9.11 ± 0.26e	75.33 ± 0.49c	2.76 ± 0.22c	23.67 ± 0.33b	15.87 ± 0.02c
**V08**	31.67 ± 1.45b	11.79 ± 0.11d	55.48 ± 0.93c	1.61 ± 0.20e	5.67 ± 0.67f	19.90 ± 0.48b	32.33 ± 0.67d	10.44 ± 0.19e	67.28 ± 2.15d	2.08 ± 0.21 g	17.33 ± 0.67d	14.56 ± 0.13d
**V09**	30.00 ± 1.53b	17.56 ± 0.78b	51.25 ± 1.52c	2.12 ± 0.14c	8.67 ± 0.88e	15.94 ± 0.34d	47.00 ± 0.58a	16.22 ± 0.62c	63.90 ± 0.99d	2.35 ± 0.22e	17.67 ± 0.33d	21.55 ± 0.87a
**V10**	41.00 ± 0.67a	19.89 ± 0.73a	91.08 ± 1.04a	1.72 ± 0.34d	16.67 ± 1.33b	12.34 ± 0.21e	41.33 ± 0.58b	18.89 ± 1.06b	119.4 ± 0.88a	3.10 ± 0.16a	23.33 ± 0.88b	10.88 ± 0.41e
**V11**	43.00 ± 1.00a	20.17 ± 0.51a	47.31 ± 1.03d	2.34 ± 0.18b	22.00 ± 0.67a	16.99 ± 0.43c	47.00 ± 1.15a	18.67 ± 0.48b	58.05 ± 1.26e	2.46 ± 0.14e	26.33 ± 1.15e	15.92 ± 0.11c
**V12**	33.00 ± 1.15b	22.37 ± 0.99a	83.74 ± 1.82a	3.28 ± 0.17a	19.00 ± 1.20a	19.77 ± 0.23b	42.00 ± 1.15b	20.11 ± 1.23a	86.49 ± 1.54b	3.05 ± 0.13a	23.67 ± 1.15b	18.38 ± 0.31b
**V13**	27.67 ± 0.58c	19.97 ± 1.39a	63.76 ± 1.21b	2.00 ± 0.03b	13.67 ± 0.33c	17.21 ± 0.26c	39.00 ± 1.20b	19.89 ± 0.67a	85.54 ± 1.24b	2.47 ± 0.09e	23.67 ± 0.67b	12.14 ± 0.14e

Means are provided with error bars. Small letter indicate significant (*p* ≤ 0.05) difference between varieties of *Cynodon dactylon* treatments. PH (plant height), SL (stolon length), LL (leaf length), LW (leaf width), NT (no of tillers), EL (electrolyte leakage)

Under drought conditions the PH and EL was significantly increased in V09, SL in V12 and 13, LL and LW in V10 and NT in V05. The least PH in V04, SL V03, LL in V05 and LW in V08, NT in V10 and EL in V01 was found (Table 3).

### Photosynthetic pigments

The Chl *a*, Chl *b* was higher in V10, total Chl and carotenoids was maximum in V04. The variety 11 showed least Chl *a*, Chl *b* in V12, total Chl in V13 and carotenoids in V03 under control treatment (Table 4). Under drought stress, Chl *a* was enhanced in V10, Chl *b* in V04, total Chl in V08 and carotenoids in V02. While, reduced Chl *a* and total Chl in V13, Chl *b* and carotenoids in V11 was observed under drought (Table 4).

**Table 4:** Photosynthetic pigments of *Cynodon dactylon* under control and drought stress

	Control				Drought			
Var.	Chl a (mg g^− 1^ FW)	Chl b (mg g^− 1^ FW)	Chl a + b (mg g^− 1^ FW)	Caro. (mg g^− 1^ FW)	Chl a (mg g^− 1^ FW)	Chl b (mg g^− 1^ FW)	Chl a + b (mg g^− 1^ FW)	Caro. (mg g^− 1^ FW)
**V01**	3.62 ± 0.04b	1.07 ± 0.01e	33.59 ± 0.74e	2.02 ± 0.03b	3.91 ± 0.10e	1.10 ± 0.01f	32.85 ± 1.32e	1.86 ± 0.23f
**V02**	2.05 ± 0.02f	1.25 ± 0.02d	44.60 ± 0.44d	1.24 ± 0.01e	4.37 ± 0.47d	2.39 ± 0.02c	56.78 ± 1.62b	4.06 ± 0.10a
**V03**	3.73 ± 0.01b	1.35 ± 0.01c	52.73 ± 0.16d	1.14 ± 0.03f	4.21 ± 0.08d	2.54 ± 0.06b	58.29 ± 0.81b	2.93 ± 0.08c
**V04**	6.03 ± 0.38a	2.23 ± 0.34a	83.94 ± 0.84a	3.38 ± 0.09a	6.24 ± 0.03b	3.87 ± 0.04a	54.74 ± 0.90b	2.03 ± 0.03e
**V05**	4.64 ± 0.13b	1.84 ± 0.03b	44.26 ± 0.54d	1.91 ± 0.01c	6.24 ± 0.20b	3.26 ± 0.02a	46.79 ± 1.50c	3.67 ± 0.11b
**V06**	2.65 ± 0.01c	1.04 ± 0.03e	38.01 ± 0.56e	1.90 ± 0.04c	3.65 ± 0.10f	1.37 ± 0.06e	51.14 ± 1.68b	2.30 ± 0.48d
**V07**	2.34 ± 0.02d	1.04 ± 0.02e	34.26 ± 0.54e	1.81 ± 0.08d	3.28 ± 0.14 g	1.27 ± 0.04e	46.70 ± 1.48c	1.81 ± 0.11f
**V08**	2.76 ± 0.09c	1.72 ± 0.13b	75.79 ± 1.45b	1.91 ± 0.01c	6.32 ± 0.06a	2.30 ± 0.03c	88.71 ± 1.04a	3.56 ± 0.07b
**V09**	2.21 ± 0.05e	1.12 ± 0.03e	47.52 ± 0.81d	1.93 ± 0.10c	3.72 ± 0.56f	1.38 ± 0.11e	51.86 ± 1.34b	2.47 ± 0.36d
**V10**	6.44 ± 0.06a	2.28 ± 0.04a	63.94 ± 1.18c	1.46 ± 0.05d	7.11 ± 0.47a	2.66 ± 0.08b	57.24 ± 1.30b	2.44 ± 0.10d
**V11**	1.41 ± 0.10 g	1.09 ± 0.02e	33.41 ± 1.08e	3.10 ± 0.16a	2.41 ± 0.09 g	0.96 ± 0.09 g	34.81 ± 1.34d	1.29 ± 0.29 g
**V12**	6.01 ± 0.31a	0.78 ± 0.07f	40.13 ± 0.73d	1.41 ± 0.04d	5.90 ± 0.14c	1.07 ± 0.32f	36.61 ± 0.56d	1.04 ± 0.37 h
**V13**	2.20 ± 0.10e	1.24 ± 0.06c	29.64 ± 0.55e	1.53 ± 0.05d	2.12 ± 0.14 g	1.84 ± 0.02d	29.21 ± 0.24f	1.17 ± 0.15 g

Means are provided with error bars. Small letter indicate significant (*p* ≤ 0.05) difference between varieties of *Cynodon dactylon* treatments. Chl a (chlorophyll a), Chl b, (chlorophyll b), Caro (carotenoids)

### Photosynthetic traits

**Fig. 1:**
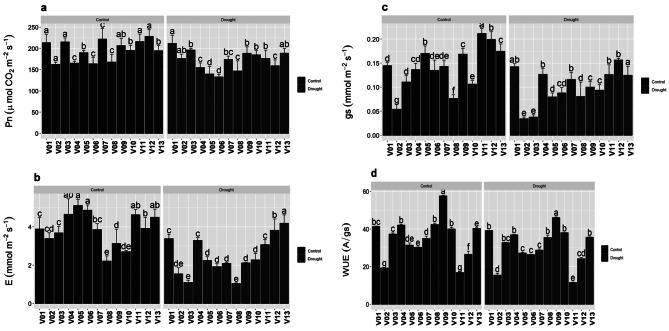
Photosynthetic traits of *Bermuda* grass verities under drought stress. Means ± SE are provided. Bars sharing same lowercase letters are not-significant (*p* ≤ 0.05) in response to control and drought conditions. (**a**) The net CO_2_ assimilation rate (*Pn*), (**b**) the transpiration rate (*E*), (**c**) the stomatal conductance (*gs*), (**d**) the water use efficiency

Net CO_2_ assimilation rate (*A*) was high in V01, V03, V11 and V12 but reduced in V08 under control. V01 high and V06 showed lower rate under drought stress as well (Fig. 1A). Transpiration rate (*E*) substantially decreased under water deficit environment in all species. Among all genotypes, a high rate was observed in V05 and V06 but lowest in V08 under control treatment (Fig. 1B). A significant higher rate was shown by V13 in water deficit conditions but gradually lowers down in V03 and V08. Stomatal conductance (*gs*) was significantly (*p* ≤ 0.05) high in V11 and less in V02 under control however it was higher in V13 and remained same in V02 in drought (Fig. 1C). Water use efficiency (WUE) decreased considerably in drought stress. Under control treatment higher rate was observed in V09 and lower in V11 hence same was observed in water deficit conditions (Fig. 1D). The relative water content was decreased under water deficit conditions. The RWC was significantly (*p* ≤ 0.05) high in V09 and less in V11 under both control and drought environment (Fig. 1E).

### Organic osmolytes

**Fig. 2:**
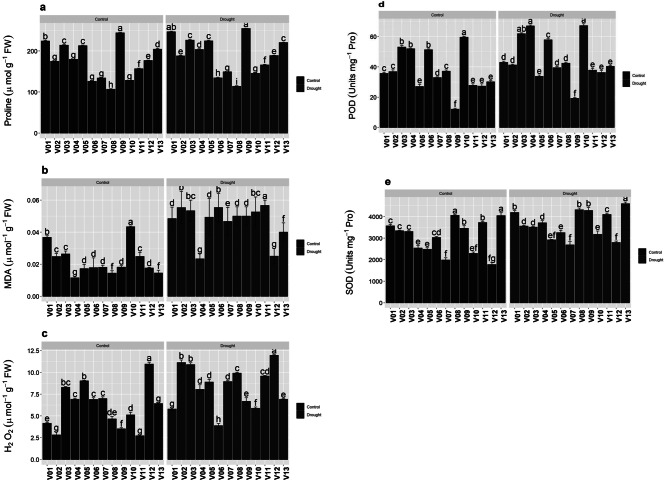
Organic osmolytes (Proline), lipid peroxidation (MDA), ROS (H_2_O_2_) and cellular antioxidants of *Bermuda* grass verities under drought stress. Means ± SE are provided. Bars sharing same lowercase letters are not-significant (*p* ≤ 0.05) in response to control and drought conditions. MDA (malondialdehyde), ROS, (reactive oxygen species), H_2_O_2_ (hydrogen peroxide), POD (peroxide dismutase), SOD (superoxide dismutase)

In organic osmolytes proline significantly (*p* ≤ 0.05) increased under drought conditions. Under control it was maximum in V09 and minimum in V08 whereas, under drought it was higher in V01, remained unchanged in V09 and V08, i.e. high and low respectively (Fig. 2A). Lipid peroxidation, Malondialdehyde (MDA) effectively increased under water deficit environment. In control it was higher in V10 but least in V04 whereas, in drought it as higher in V11 but minimum in V04 and V12 under drought (Fig. 2B). Reactive oxygen species (ROS) as (H_2_O_2)_ significantly (*p* ≤ 0.05) enhanced in drought stress. High rate of H_2_O_2_ was observed in V12 and low in V11 in normal conditions, however remained unchanged under drought i.e. higher in V12 but least in V06 (Fig. 2C).

### Cellular antioxidants

Enzymatic antioxidants peroxide dismutase (POD) and superoxide dismutase (SOD) was dramatically increased in all genotypes under drought conditions. POD showed a considerable increase in drought stressed environment in all genotypes; in normal conditions it was higher in V10 but least in V09 and in water deficit it increased in V04 and V10 however, lower POD concentration observed in V09 (Fig. 2D).

SOD was higher in V08 and V13 but least in V07 and V12 in normal conditions whereas, in drought stress it increased in V13 but remained unchanged i.e. least in V07 and V12 (Fig. 2E).

### Expression level of genes present in drought responses

**Fig. 3:**
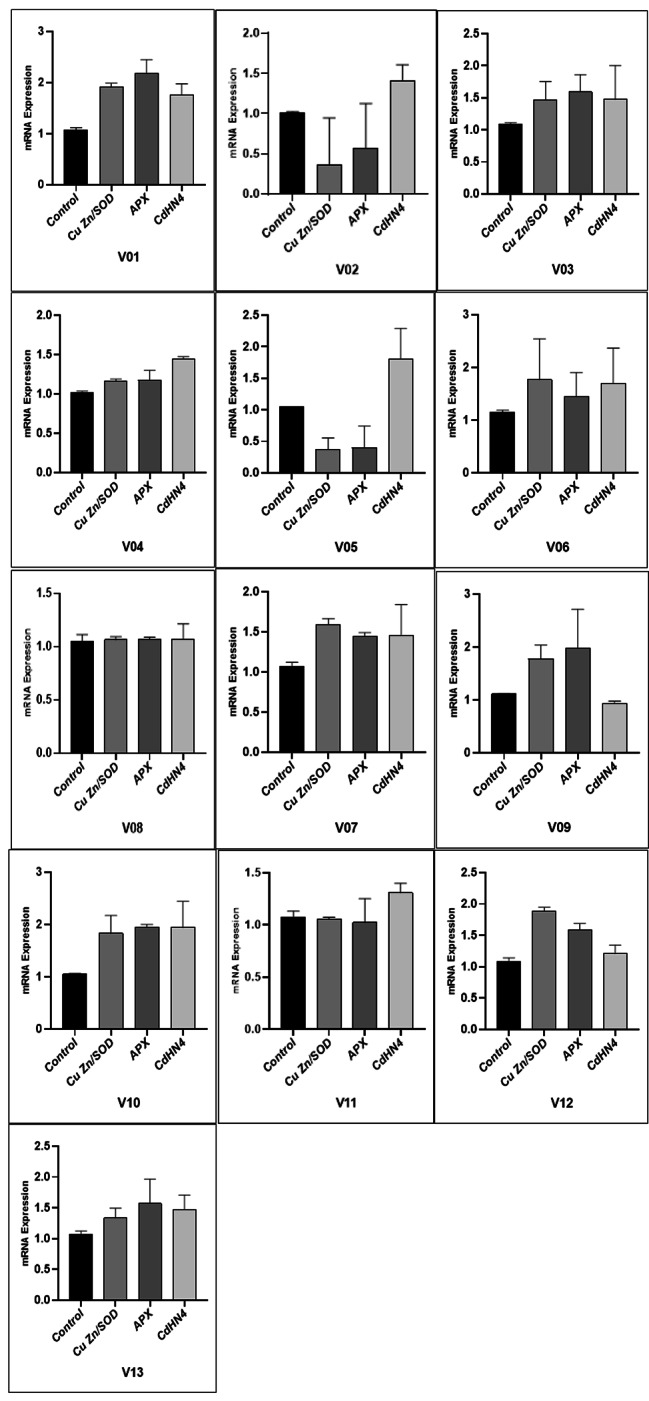
Gene expression in response to drought conditions in Bermuda grass verities. mRNA (messenger RNA)

The three genes in which two are responsible to antioxidant activation and one dehydrin were studied to find transcriptional responses under control and drought stress. All genes showed a differential expression level in all genotypes. The *Cu/ZnSOD* gene was upregulated in all the genotypes except V02 and V05 but in V08 and V11 showed no expression (Fig. 3). The higher expression level was observed in V01, V10 and V12. The *APX* gene was upregulated in most of the species but downregulation was observed in V02 and V05. The V08 and V11 showed no gene expression under stress. The highest level of expression was shown by V01 and V09. The dehydrin gene named *CdDHN4* showed significant upregulation expression in all genotypes, whereas in V08 no expression was seen. Higher expression level was observed in V05 and V10 (see Fig. 3).

### Multivariate analysis

#### Principal component analysis

**Fig. 4:**
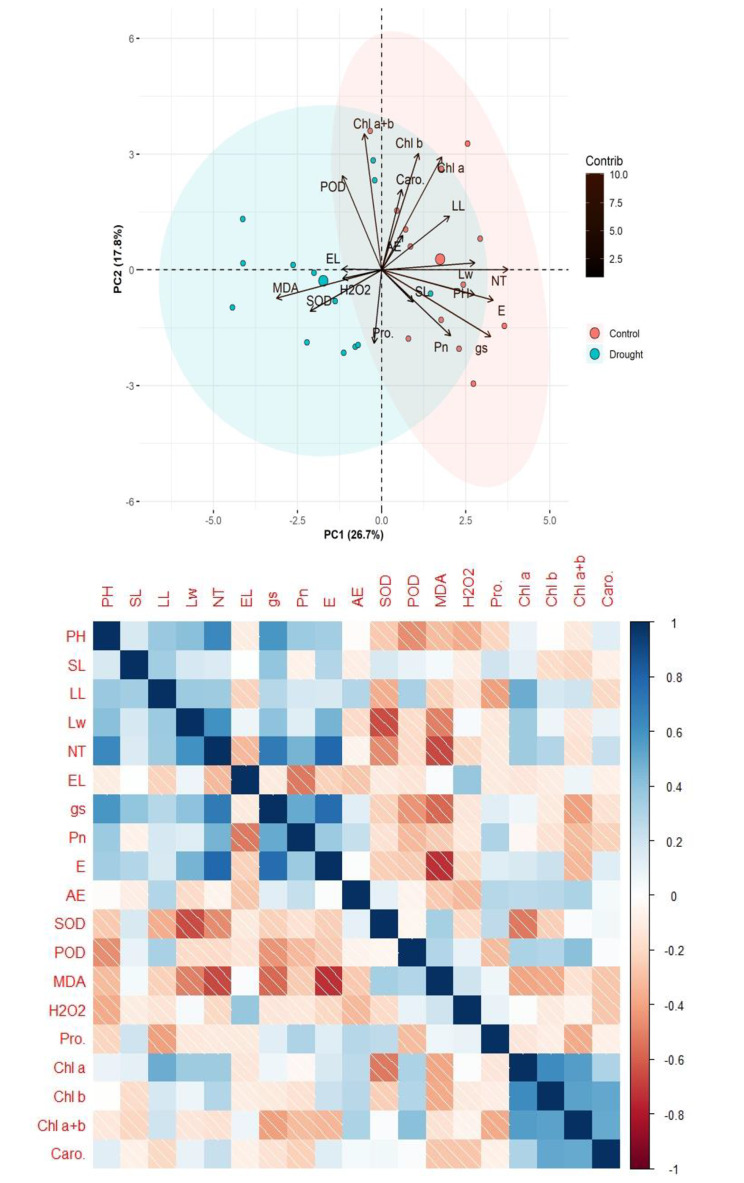
PCA biplot and correlation matrix of morphology, organic osmolytes and gas exchange and photosynthetic pigments of *Cynodon dactylon* in response to control and drought treatments. PH (plant height), SL (stolon length), LL (leaf length), LW (leaf width), NT (no of tillers), EL (electrolyte leakage). Chl a (chlorophyll a), Chl b, (chlorophyll b), Caro (carotenoids). Net CO_2_ assimilation rate (*Pn*), transpiration rate (*E*), stomatal conductance (*gs*), (WUE) water use efficiency. MDA (malondialdehyde), ROS, (reactive oxygen species), H_2_O_2_ (hydrogen peroxide), POD (peroxide dismutase) and SOD (superoxide dismutase)

Principal component analysis was constructed for the morphology, organic osmolytes, gas exchange and photosynthetic pigments of *Cynodon dactylon* in response to control and drought treatments (Fig. 4). The PCA biplot showed an influential response with a cumulative contribution of 44.5% variations. The ROS (H_2_O_2_), MDA, and SOD were loaded toward drought ellipsed with higher negative eigenvalues (> 3). These traits were closely associated with *Cynodon dactylon* verities as DV5, DV13, DV9 and DV7. The *C.dactylon* verities are strongly associated with DV1:DV9 and DV11. The photosynthetic pigments Chl *a + b*, and Chl *b* showed a strong association with higher positive eigenvalues (> 3) and excelled toward PC2. These traits also diverged to the drought ellipse group and strongly exhibited drought tolerance in DV4, CV4 and DV 10. The Chl *a* and Carotenoids also exhibit the same pattern of drought tolerance with a positive eigenvalue of > 2 and grouped with CV4 and CV8. The EL and AE were significantly associated with varieties of *C.dactylon* DV3, DV6, CV3 and CV5. The antioxidant enzyme significantly enhanced in drought conditions and empowered the significantly higher eigenvalue of the eigenvectors and was closely associated with DV4 and DV10. The SL, LW, PH, NT, E, Pn and gs responded positively and loaded toward the PC1 in a reducing pattern. These traits also shown association with *C.dactylon* as NT: CV11, PH: CV7, E: CV9, Pn: CV13 and SL: CV7 (Fig. 4A).

### Pearson correlation matrix

The Pearson correlation matrix drew a significant (*p* ≤ 0.05) correlation for the morphology, organic osmolytes gas exchange and photosynthetic pigments of *Cynodon dactylon* in response to control and drought treatments (Fig. 4B). The growth traits PH, SL, LL, LW, NT and NT negatively skewed with SOD, POD, MDA, H_2_O_2_ and Proline, while positive skewing with photosynthetic pigments. The PH, LW and NT were significantly and positively correlated with the gas exchange traits gs, Pn, and E. The WUE (A/E) was positively related with Pro, Chl a, b, a/b and carotenoids. The ROS showed a strong negative influence on photosynthetic and antioxidant enzymes (Fig. 4B).

### Clustered heatmap and dendrogram

**Fig. 5:**
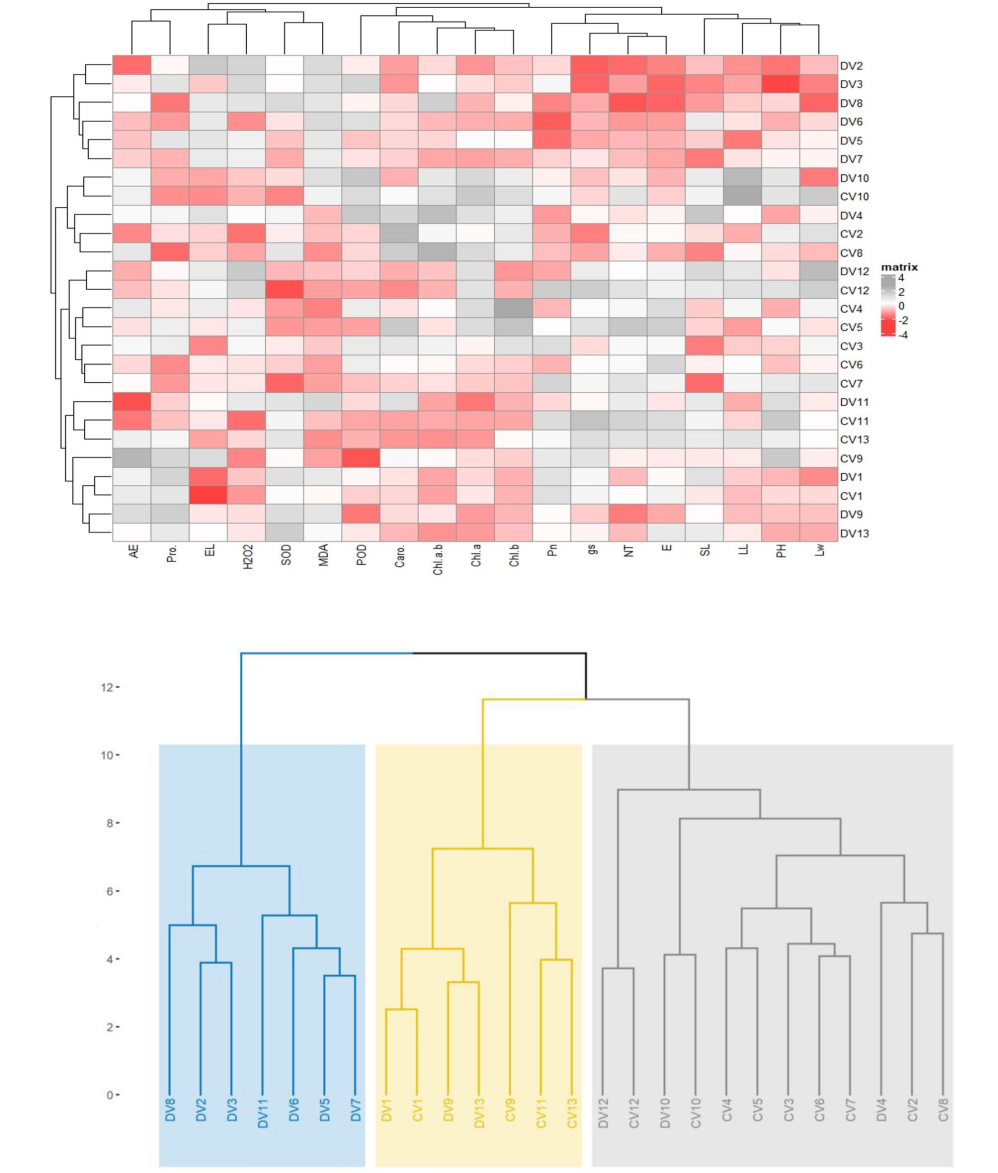
Clustered heatmap of morphology, organic osmolytes, gas exchange and photosynthetic pigments of *Cynodon dactylon* and hierarchical dendrogram in response to control and drought treatments. C (control), D (drought), PH (plant height), SL (stolon length), LL (leaf length), LW (leaf width), NT (no of tillers), EL (electrolyte leakage). Chl **a** (chlorophyll a), Chl **b**, (chlorophyll b), Caro (carotenoids). Net CO_2_ assimilation rate (*A*), transpiration rate (*E*), stomatal conductance (*gs*), (WUE) water use efficiency. MDA (malondialdehyde), ROS, (reactive oxygen species), H_2_O_2_ (hydrogen peroxide), POD (peroxide dismutase) and SOD (superoxide dismutase)

A clustered heatmap was constructed to demonstrate the effect of drought on the morphology, organic osmolytes, gas exchange and photosynthetic pigments of *Cynodon dactylon*. The LW positively linked with DV12, CV12, CV10, however negative influence with Dv1, DV8, DV3, DV2 and DV10. The PH negatively corresponded with DV2 and DV3, while showing a positive response with CV11 and CV9. The LL and SL have shown a negative trend with DV8, DV3, DV2 and positive DV12 and CV12. The Pn, gs and NT negatively corresponded with DV2, DV3, DV8, DV6 and DV5. The Chl *b* showed a positive turn with CV4 and CV5, while the Chl a showed a positive relation with DV4, DV12 and DV10. The POD was more positively influenced in DV10 and DV4, while negatively in CV9 and DV9. The antioxidant SOD corresponded positively with DV9 and DV13, and negatively related with CV7 and CV12. The AE was positively associated with CV9 and DV9 (Fig. 5A).

A hierarchical dendrogram was constructed to show a clustering between varieties of *Cynodon dactylon*. The DV1, DV2, DV3, DV11, DV6, DV5, DV7 strongly clustered under drought conditions. The DV1, CV1, CV9, CV13, CV9, CV11, and CV13 were strongly grouped. The DV12, CV12, DV10, CV10, CV4, CV5, CV3, CV6, CV7, CV3, DV4, CV2, CV8 were clustered with each other (Fig. 5B).

## Discussion

Drought is a key factor limiting bermudagrass growth and utilization. The plant’s response to drought depends on stress severity, duration, variety, and developmental stage [27]. Turfgrasses have three main drought-resistance approaches: drought tolerance, avoidance, and escape which help them to survive during prolonged drought [28]. These approaches are genetically under the control of several physio-biochemical characteristics at cell, tissue, organ and whole-plant level as well. Thus, finding a way of improvement of the drought tolerant and resistant genotypes is important in turfgrass [3]. In our study, the response mechanisms of different bermudagrass accessions were evaluated in order to find the most tolerant genotype for water deficient conditions. Drought stress limits plant growth, particularly during the early stages of their life cycle by affecting cell elongation and enlargement [29].

In our study, growth traits including growth habit (vertical), decreased plant height, stolon length, reduced tiller, leaf length and width may results in lower evapotranspiration rate, to save maximum water content which would delay dehydration of the grass [30]. Our growth traits results are similar to findings of [31] that explains the higher root/shoot density, in terms of deep rooting with more tiny root hairs, but reduced plant height, stolon length are direct selection of drought resistance and recovery from damage. Our V10 and V11 optimally responded better under water deficit conditions by securing more growth traits as PH, LL and LW, while the varieties V12 and V13 have maximum SL under drought stress.

Drought stress precisely induces oxidative stress in grass by the overproduction of ROS and MDA. It decreases the relative water content by disturbing the osmotic potential and increased the ion leakage. RWC (relative water content) of leaf reflects water status of plant, it shows the equilibrium between water uptake by tissues of leaf and transpiration rate. whereas, ion leakage is the electrolytes leaked from a tissue that reflects the injury of the cell membrane [32]. Malondialdehyde (MDA) is one of the final product in the cells peroxidation of polyunsaturated fatty acids and is an index of the oxidative injury of plants. MDA level is basically a marker of oxidative damage and the antioxidant level in plant [25]. ROS are actually a byproduct of cellular oxidative metabolisms that plays an important role in cell differentiation, signaling and death. ROS are mainly, H_2_O_2_ (hydrogen peroxide) content, OH (hydroxyl ion) and ^1^O_2_ (singlet oxygen). Plants protect themselves by osmotic regulation and producing proficient antioxidative systems for ROS scavenging. Osmotic adjustment is possible by accumulation of osmolytes and osmoprotectant like soluble carbohydrates, soluble sugars and amino acids particularly proline and glycine betaine which plays a vital role against drought stress. Within plant cells an increased proline content may increase the water potential which may results in more water uptake, which helps to maintain a high relative water content (RWC) [25]. Proline has strong hydrophilic ability which has a protective role in cell membrane structure. It also has role in scavenging of singlet oxygen and hydroxyl ion [33]. On other way, proline stimulates the enzymatic antioxidants to remove ROS [34]. The maximum proline accumulation was observed in V01 and V09 under water scarcity.

Turfgrasses contain enzymatic antioxidants as SOD, POD or CAT and non-enzymatic antioxidants comprising carotenoids and flavonoids that are also crucial for ROS stability in grass plant [35]. The superoxide dismutase is a basic enzyme for maintaining regular physiological processes and managing with oxidative damage by quickly converting O_2_^−^ to O_2_ and H_2_O_2_ [36]. In our experiment higher MDA content, fatty acid composition, increased antioxidant enzyme (SOD, POD) and increased non-enzymatic antioxidant activity with similar [37]. The MDA contents significantly lowered in V04 and V12 which showed more tolerance to drought. The least amount of H_2_O_2_ was assessed in V01, V06, V09 and V10 and antioxidants enhanced in V09 and V10.

In our experiment, under moderate drought, Pn (net assimilation rate) reduced very fast along with the reduction in gs (stomatal conductance), proposing that the earlier photosynthesis limitation in *C. dactylon* was because of rapid stomatal closure. Our results are similar with the findings of [38] a relatively less stomatal conductance was observed in *C. dactylon* in earlier drought stage, Moreover, reduction in evapotranspiration rate, transpiration sensitivity, and results in reduction of water loss by increasing stomatal resistance. Previous findings observed that moderate drought can reduce stomatal conductance, limiting CO_2_ diffusion to intercellular spaces and ultimately affects Pn rate. Under earlier drought, it has been experienced that stomata has a leading role in regulating the reduced net CO_2_ uptake. This leads to a reduction in CO_2_ concentrations inside the leaf. During previous drought conditions, it has been observed that stomata play a crucial role in regulating the decrease of net CO_2_ uptake. This leads to a reduction in the concentration of CO_2_ inside the leaf. Additionally, the limitations to CO_2_ uptake caused by stomatal closure can create an imbalance between photochemical activity at PSII and electron requirements for photosynthesis. This imbalance results in overexcitation and subsequent photoinhibitory damage to PSII reaction centers [39]. It is clear that a correlation exists between stomatal conductance and leaf water potential [40].

Water scarcity can cause a decline in non-stomatal mechanisms including chlorophyll synthesis, change in function and structure in chloroplasts [41]. The WUE increased in V09 and V10 while, the photosynthetic performance was efficient in V01 and V02 in terms of Pn, significant transpiration reduction occurred in V03 and V08, least stomatal conductance occurred in V02, and V03 under drought stress. As reduced photosynthesis is might be due to change in leaf structure, decreased chlorophyll pigments as *a*, *b* and total chlorophyll content [25]. One of reason could be degradation of chlorophyll which occurred under given conditions because a positive correlation observed in photosynthesis rate and chlorophyll content. On the other hand, the reason of reduction of chlorophyll content is degradation of chloroplast membrane, unnecessary swelling, distortion of middle lamellae, and lipid droplets appearance. Our results accordance with [36]. According to our results, photosynthetic rate and pigments reduced with severity of drought stress. The V10 also exhibited maximum photosynthetic pigments as Chl *a*, Chl *b* and Chl *a + b* concerning its counterparts.

However, drought-related genes have been identified, but their functions and regulation pathways are unclear and require further study. At present, the natural variations between different *C.dactylon* genotypes provide effective strategies to explore the molecular, proteomic, and metabolic approaches in drought stress response. Total 39 proteins involved in glycolysis, tricarboxylecacid, N-metabolism and photosynthetic pathways in drought-stressed bermudagrass [10]. A dehydrin gene called *CdDHN4* in bermudagrass is a member of late embryogenesis abundant-II family with 9 ∼ 200 kDa molecular weight. A highly hydrophilic protein, that plays a significant role in cell protection from dehydration damage. It enhances drought tolerance via an ABA-dependent signaling pathway by protecting the membrane and protein structure by acting as a chaperone [42]. The Q-PCR results indicated that expression level of *CdDHN4* increased with progressive drought. Our findings are similar with dehydrin expression report by [43].

Under oxidative stress the antioxidant enzyme activities such as SOD, POD, and APX significantly increased in *C.dactylon* [13]; therefore, to investigate the expression level of responsible genes for encoding the following enzymes was necessary. There are a number of genes encoding SODs, each of which producing an isozyme. Additionally, these SOD isoenzymes are specifically localized in different organelles such as chloroplasts, mitochondria, peroxisomes, and cytosol [44]. *Cu/ZnSOD* is a gene that is present in both the chloroplasts and the cytosol of higher eukaryotes including bermudagrass [45]. In our study, some ROS scavenging gene’s *CuZn/SOD* (copper zinc superoxide dismutase) and *APX* expression was observed and results was similar to [13]. Q-PCR results showed higher expression of these genes under water deficit. The findings indicate that there exists a close correlation between the activity level of antioxidant enzymes and the expression of their corresponding genes. In general, V01 along longitude and V10 along latitude showed significantly higher tolerance; while, V04 along longitude and V13 along latitude gradient showed tolerance susceptibility under water deficit in comparison with others.

Furthermore, we evaluated a correlation of all above measured traits. In these traits, the RWC and MDA showed a highest positive correlation with EL. The relation among RWC and EL showed that the status of plant water regulation is very significant for maintenance of plant cell membrane. Our analysis revealed that oxidative stress during drought has severely affected the cell membrane stability, resulting in injury to cells by ROS production and the plant’s antioxidant defense system. Proline, as an osmolyte, plays a key role in eliminating singlet oxygen and hydroxyl ions, protecting antioxidant enzymes (SOD, POD) and their relevant genes. Proline also facilitates osmotic adjustment, which reduces drought-induced cell injury, lipid peroxidation, and increases plant survival rate.

### Conclusions and future perspectives

In our study the effect of drought was assessed in 13 different *Cynodon dactylon* varieties. The *Cynodon dactylon* genotypes responded differentially under drought conditions by securing better growth traits in terms of more PH, LL, LW and SL. The high photosynthetic efficiency in the form of Pn, reduced transpiration and least stomatal conductance by accumulating more Chl *a*, Chl *b* and Chl *a + b* content observed. The results revealed, high WUE, increase in organic osmolytes, lower ROS and MDA contents in tolerant varieties. Drought conditions not only showed the expression of dehydrin *cdDHN4* gene but also up-regulated the antioxidant-related *APX*, *CuZn/SOD* genes by enhancing the antioxidant activities. The present work results demonstrated that various varieties of the *Cynodon dactylon* have the potential to mitigate harsh drought conditions and can easily grow in water deficit extremes. The newly introduced species can be an excellent germplasm for future studies on drought tolerance mechanisms and pathways involved in it. The genes with up-regulated expression can be transformed into crops to meet the world food scarcity.

## Data Availability

No datasets were generated or analysed during the current study.

## Abbreviations

CV: Control variety
DV: Drought variety
LSD: Least significant difference
PH: Plant height
SL: Stolon length
LL: Leaf length
LW: Leaf width
NT: Number of tillers
EL: Electrolyte leakage
Chl: Chlorophyll
WUE: Water use efficiency
MDA: Malondialdehyde
ROS: Reactive oxygen species
H_2_O_2,_: Hydrogen peroxide
SOD: Superoxide dismutase
POD: Peroxide dismutase
PCA: Principle component analysis
